# Supplementary material for: Artificial Neural Network Language Models Predict Human Brain Responses to Language Even After a Developmentally Realistic Amount of Training
Source: Neurobiol Lang (Camb). 2024 Apr 1;5(1):43–63. doi: 10.1162/nol_a_00137 (PMC11025646; doi:10.1162/nol_a_00137)
Supplement: Supplementary file 1 [file nol-5-1-43-s001.pdf]

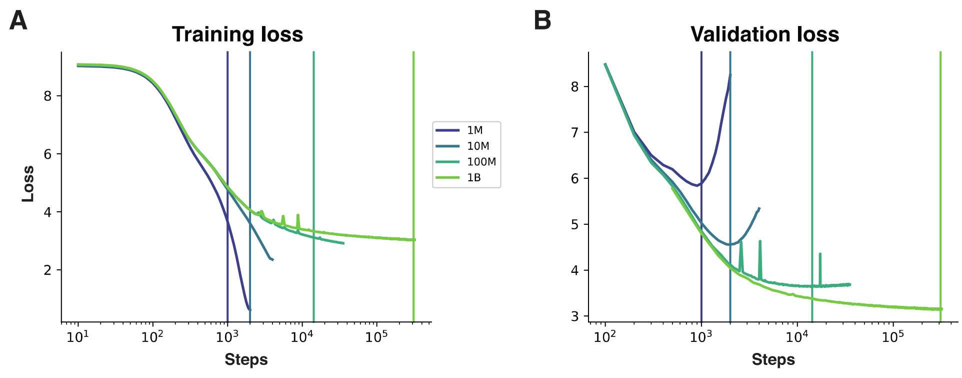

Supplementary Figure 1. Training and validation loss for the models in Experiment 1. For all models, a training batch size of 64 was used (each element in a batch is a sequence of 1,024 tokens). The training was done with a standard Adam optimizer (LR: 0.0006, beta: [0.9,0.999], eps:1e-8), and cosine learning rate (starting at 0.0006 and decaying over 320000 training steps), with gradient accumulation and clipping, attention and hidden dropout ratio of 0.1, and no weight decay. **A.** Training loss: as expected, all models show a drop in loss with additional training, and for models trained on smaller datasets, the rate of learning is faster. **B.** Validation loss: as expected, all models show a drop initially, and models trained on smaller datasets reach their minimum loss earlier in the training process and have higher values (corresponding to worse performance) than models trained on larger datasets. In both A and B, the vertical lines mark the step with the minimum validation loss (best model performance). The representations for the steps shown in B were used for testing against the human benchmarks.

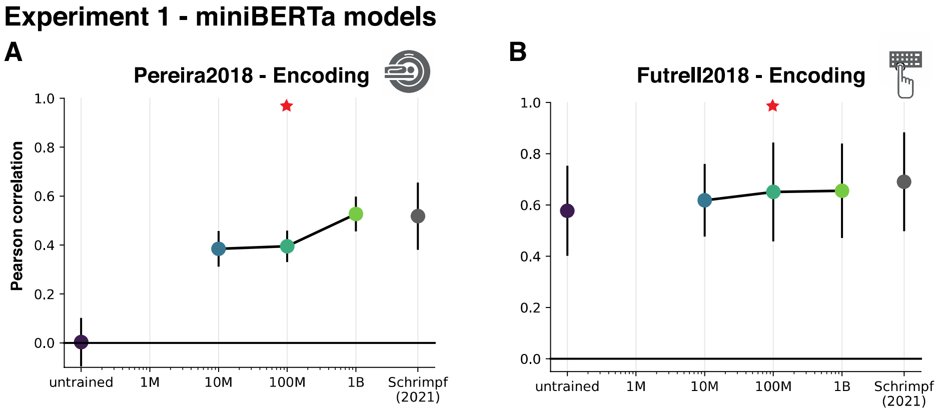

Supplementary Figure 2. Model performance on the human benchmarks for the miniBERTa models as a function of training in Experiment 1. Performance (normalized predictivity) of the best-performing layer, as reported in Schrimpf et al. (2021) for the RoBERTa model, in predicting language-responsive voxels' activation in the Pereira2018 fMRI benchmark (A), and reading times in the Futrell2018 behavioral benchmark (B) (see **Supp. Figure 3** for the results for GPT-2 for the Futrell2018 benchmark). The results are shown for i) an untrained model (black dots; the Gaussian initialization; see Methods for details); ii) three models trained on datasets of different size (10M, 100M, and 1B words) (teal-to-green dots connected by a line; the model trained on a developmentally plausible amount of data—100M—is marked with a red asterisk); and iii) a fully trained model, as reported in Schrimpf et al. (2021) (grey dots). As in the main figures, we computed a median score across participants and divided it by an estimated ceiling value to get a normalized score, and we computed a median absolute deviation over participants for use as error bars.

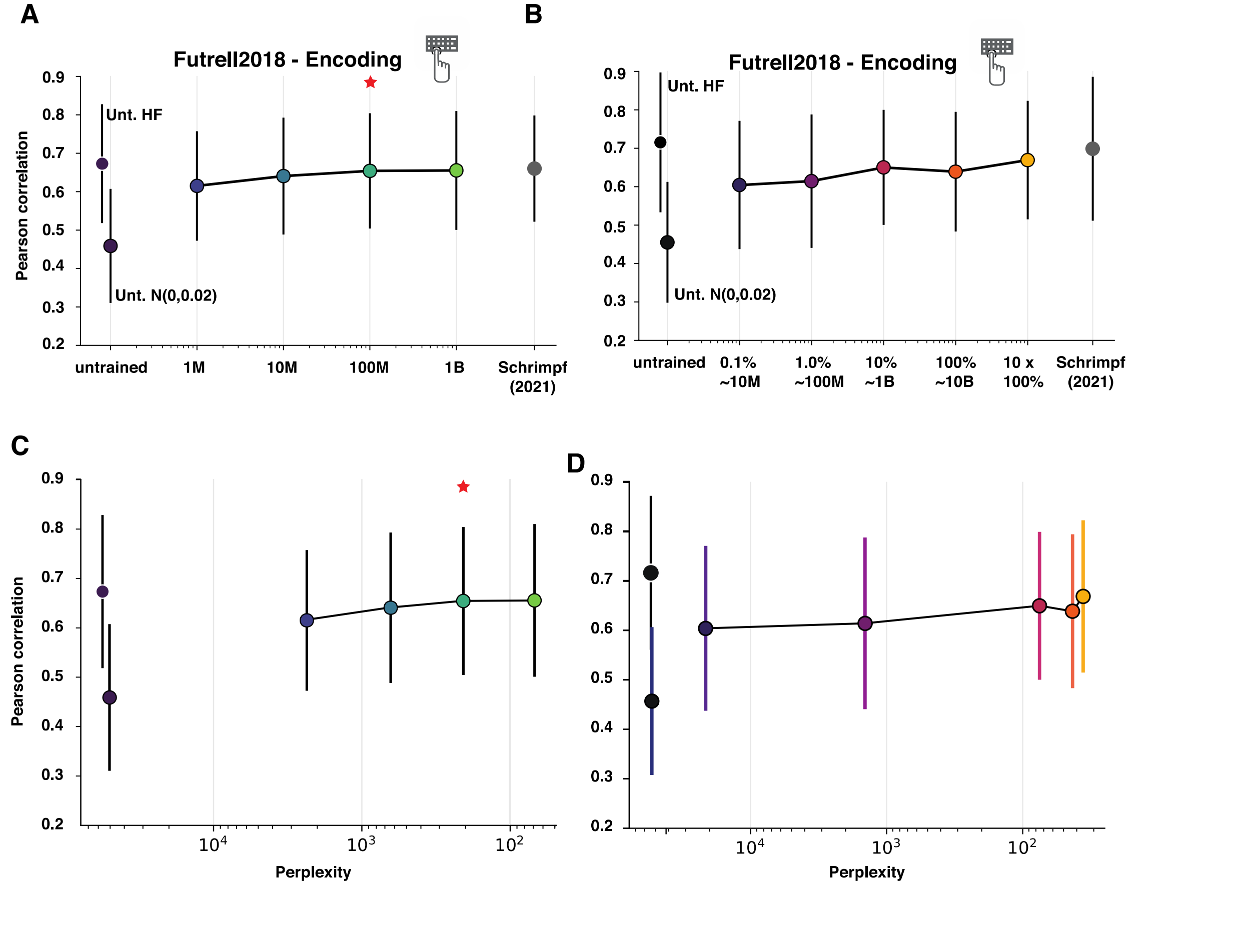

Supplementary Figure 3. Model performance on the behavioral (Futrell2018) benchmark as a function of training.

A. Performance (normalized predictivity) of the best-performing GPT-2 layer, as reported in Schrimpf et.al. (2021), in predicting reading times in the Futrell2018 benchmark. The results are shown for i) two versions of an untrained (Unt.) model (initialized in two different ways: Unt. N(0,0.02) corresponds to the untrained model initialized with a mean of 0 and a standard deviation of 0.02, and Unt. HF corresponds to the untrained model initialized with the Hugging Face parameters; see Methods) (black dots); ii) four models trained on datasets of different sizes (1M, 10M, 100M, and 1B tokens) (blue-to-green dots connected by a line; the model trained on a developmentally plausible amount of data—100M—is marked with a red

asterisk); and iii) a fully trained model, as reported in Schrimpf et al. (2021) (grey dots). As in the main figures, we computed a median score across participants and divided it by an estimated ceiling value to get a normalized score, and we computed a median absolute deviation over participants for use as error bars.

B. Performance of the last GPT-2 layer in predicting reading times in the Futrell2018 benchmark. The results are shown for i) two versions of an untrained model (initialized in two different ways, as in A; see Methods) (black dots); ii) a model trained on a large dataset examined at different points during the training (0.1%, 1.0%, 10%, 100%, and 10*100% of training steps) (purple-to-yellow dots connected by a line); and iii) a fully trained model, as reported in Schrimpf et al. (2021) (grey dots).

C. The relationship between perplexity, i.e., the model's ability to predict the next token in an independent dataset (wikitext-103-raw-v1), shown on the x-axis, with lower values corresponding to better performance, and model performance in predicting human reding times in the Futrell2018 benchmark. The results are shown for i) two versions of an untrained model (initialized in two different ways, as in A; see Methods) (black dots); and ii) four models trained on datasets of different sizes (1M, 10M, 100M, and 1B words) (blue-to-green dots connected by a line; the model trained on a developmentally plausible amount of data—100M—is marked with a red asterisk).

D. The relationship between perplexity and model performance in predicting reading times in the Pereira2018 benchmark. The results are shown for i) two versions of an untrained model (initialized in two different ways, as in A; see Methods) (black dots; see **Figure 2** caption for details); and ii) a model trained on a large dataset examined at different points during the

training (0.1%, 1.0%, 10%, 100%, and 10*100% of training steps) (purple-to-yellow dots connected by a line).

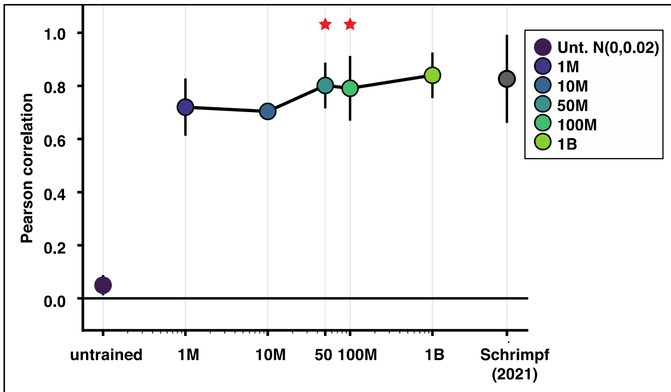

Supplementary Figure 4. Model performance on the fMRI (Pereira2018) benchmark as a function of training, including a model trained on 50M words (the data for the models other than the 50M model are identical to **Figure 2A**). Performance (normalized predictivity) of the best-performing GPT-2 layer, as reported in Schrimpf et.al. (2021), in predicting language-responsive voxels' activation in the Pereira2018 fMRI benchmark. The results are shown for i) an untrained model (initialized with a Gaussian; see Methods); ii) five models trained on datasets of different size (1M, 10M, 50M, 100M, and 1B words) (blue-to-green dots connected by a line; the models trained on developmentally plausible amounts of data—50M and 100M—are marked with a red asterisk); and iii) a fully trained model, as reported in Schrimpf et al. (2021) (grey dots). As in Figure 2, we computed a median score across participants and divided it by an estimated ceiling value to get a normalized score, and we computed a median absolute deviation over participants for use as error bars. The models trained on 50M, 100M and 1B words exhibit fMRI response predictivity that is similar to that of the fully trained GPT-2 model, with no significant differences in predictivity values ($ps>0.05$). In contrast, the predictivity of the untrained model and the models trained on 1M and 10M words is significantly below the predictivity of the fully trained model (p-values: <0.0001, $p=0.001$, and $p=0.003$, respectively).

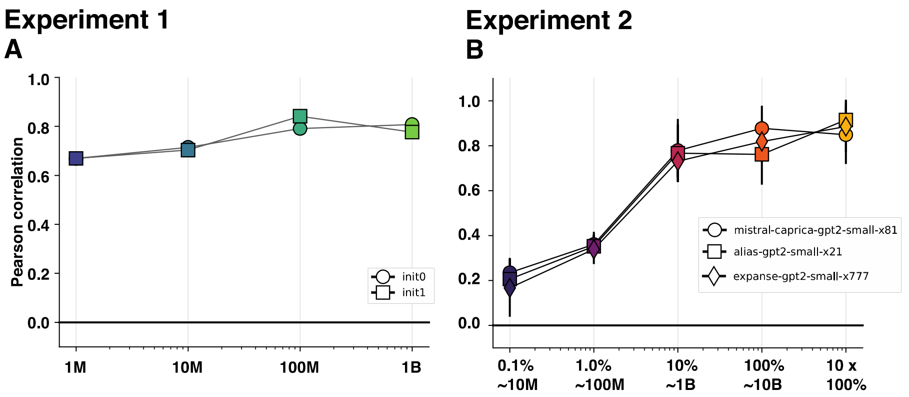

Supplementary Figure 5. Robustness of the critical results to seed choice during model initialization. Model initialization seeds have been shown to affect model performance (e.g., Mehrer et al., 2020). Here, we show that the main results (reported in Figure 2A,C) are robust the seed choice. For Experiment 1, two different seeds were used; for Experiment 2, three different seeds were used.

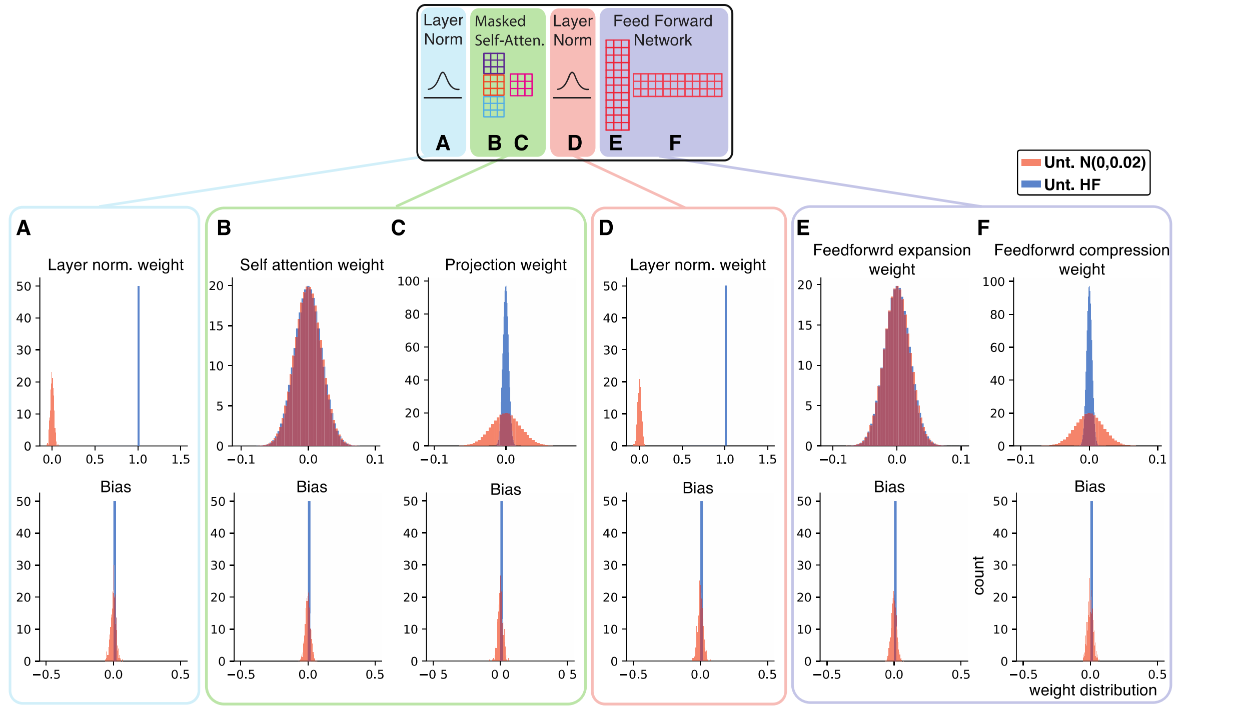

Supplementary Figure 6. A detailed comparison of the two model initializations for the untrained GPT-2 models (for a sample layer (layer 1)). The distribution of weight (top row) and bias (bottom row) values for the first layer normalization (A), two operations in self attention (B-C), the second layer normalization (D), two operations in feedforward processing (E-F) for two versions of an untrained model: Unt. N(0,0.02) corresponds to the untrained model initialized with a mean of 0 and a standard deviation of 0.02, and Unt. HF corresponds to the untrained model initialized with the Hugging Face parameters (see Methods).

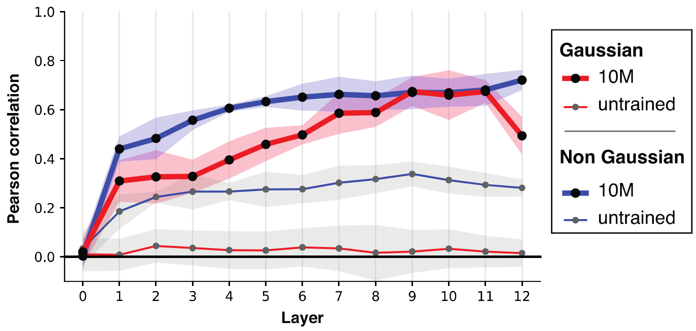

Supplementary Figure 7. Effects of differences in model initialization on the performance of an untrained GPT-2 model and a GPT-2 model trained on the 10M words on the Pereira2018 fMRI benchmark across layers.

Performance (normalized predictivity) in predicting the Pereira2018 fMRI benchmark. The models shown with blue lines were initialized with the Hugging Face parameters; the models shown with red lines were initialized with a Gaussian distribution of weights with a mean of 0 and a standard deviation of 0.02 (see Methods and **Supp. Figure 6**). The untrained model initialized with a Gaussian distribution performs close to 0 across layers. In contrast, the untrained model initialized with the Hugging Face (non-gaussian) parameters achieves ~0.4 predictivity for some layers. After training, both models reach a similar level of predictivity for their later layers (layers 9-11). We also observed a longer training time for Gaussian initialization compared to hugging-face initialization.

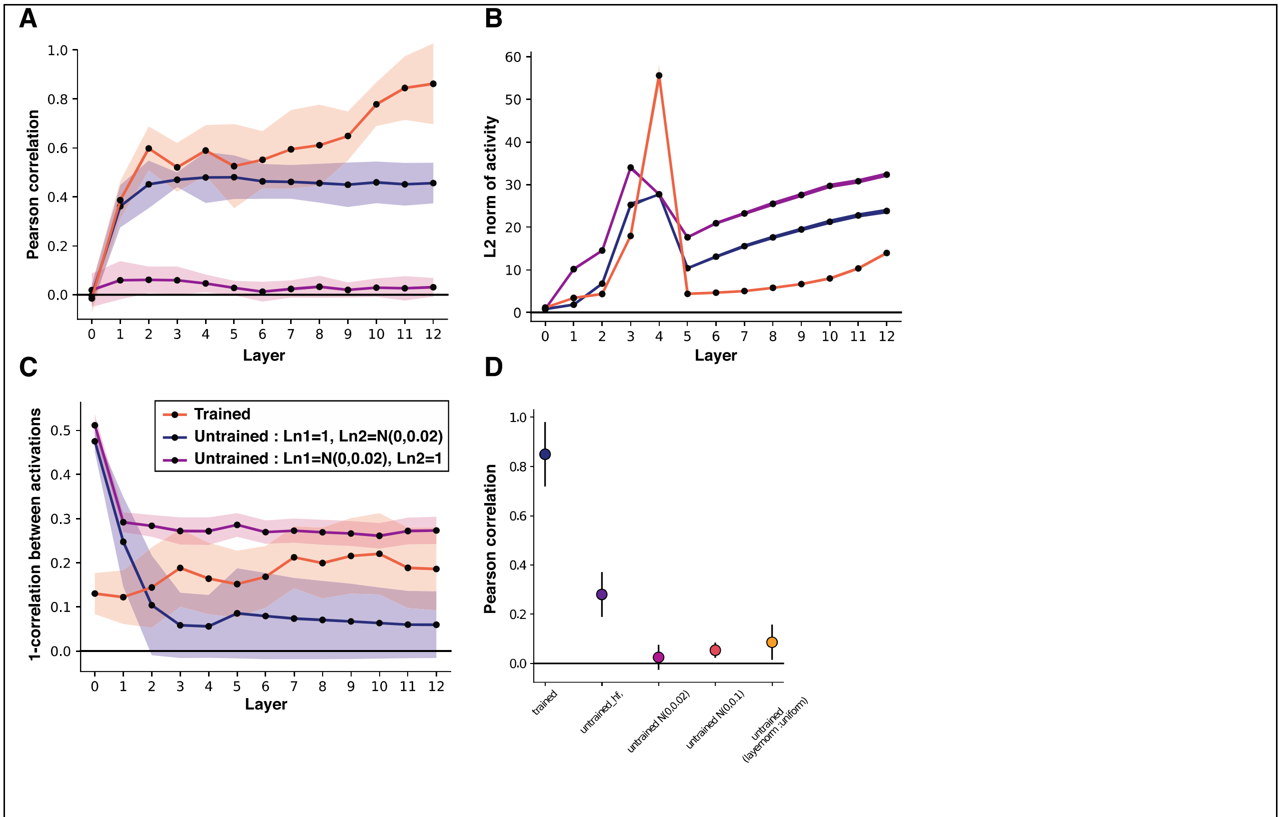

Supplementary Figure 8. Effects of differences in model initialization on the performance of untrained models on the Pereira2018 benchmark across layers.

Here, we created two versions of an untrained model that both use a Gaussian weight distribution for the self-attention and feedforward processing components of the model but differ in the weights they use for layer normalization. In particular, in one model (the dark blue line), the first layer normalization (Ln1) was set to 1 (as in the Hugging Face initialization), and the second layer normalization (Ln2) was set to a Gaussian distribution, and in the other model (the maroon line), Ln1 was set to a Gaussian distribution, and Ln2 – to 1 (as in the HF initialization). The third line (orange) corresponds to a trained model (initialized with the standard HF parameters) and is included here for comparison.

A. Performance (normalized predictivity) in predicting the Pereira2018 fMRI benchmark. The model with Ln1=1 exhibits higher performance compared to the model with Ln2=1, which suggests that the first layer normalization plays a larger role in contributing to above-zero performance for untrained models on the human benchmarks.

B. Amplitude of model activation. The two untrained models show a similar level of activation to each other (and, in some layers, to the trained model), which suggests that the difference in performance between them is not due to the lack of activity propagation across layers in the model where Ln2=1.

C. Similarity of model representations among the sentences in the Pereira2018 benchmark (higher values correspond to lower similarity). The representations appear to be more similar in the model where Ln1=1 (compared to the model where Ln2=1 or compared to a trained model), which suggests that setting Ln1 to 1 effectively removes stimulus-specific encoding and may explain the above-zero predictivity of neural responses for the untrained model initialized with the HF parameters.

D. Performance of a trained model and several versions of untrained models in predicting the Pereira2018 fMRI benchmark. The untrained models include a model initialized with the standard HF parameters, two models initialized with a Gaussian distribution that vary in the size of the standard deviation (0.02, 0.1), and a model where all LayerNorm weights are set to be a uniform distribution between 0 and 1 (all positive values). The model initialized with the HF parameters shows higher predictivity than the other untrained models, but the size of the standard deviation in the Gaussian initialization does not strongly affect performance (cf. (Rohde & Plaut, 1999)). Finally, the model initialized with a uniform distribution (of positive

values) for LayerNorm weights performs close to zero, similarly to the model with a Gaussian initialization.

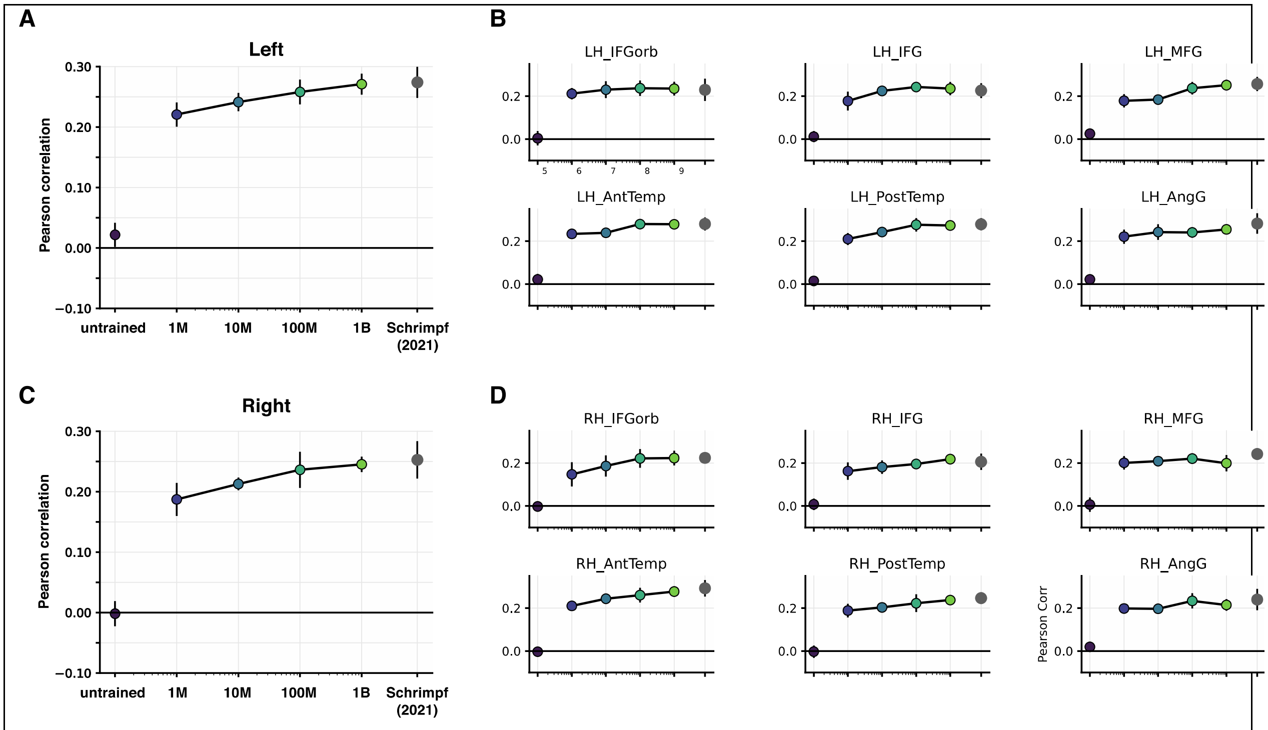

Supplementary Figure 9. Model performance on the fMRI (Pereira2018) benchmark as a function of training in the left vs. right hemisphere separately (A and C), and in the individual regions of interest within each hemisphere (B and D). Performance of the best-performing GPT-2 layer, as reported in Schrimpf et.al. (2021), in predicting language-responsive voxels' activation in the Pereira2018 fMRI benchmark for the left hemisphere (A) and right hemisphere (C) overall and broken down by functional ROI (B: left hemisphere (LH) fROIs; D: right hemisphere (RH) fROIs). Note that we here report *raw* predictivity values (cf. the normalized values reported in all other figures) so as to be able to more meaningfully compare between hemispheres and among ROIs without the complication of different ceiling values across regions. The fROIs are identified with an independent localizer, as described in Methods. The general pattern of results (presented in main Figure 2) holds across

hemispheres (although predictivity is higher in the LH, in line with other work; e.g., Schrimpf et al., 2021; Tuckute et al., 2024) and fROIs.

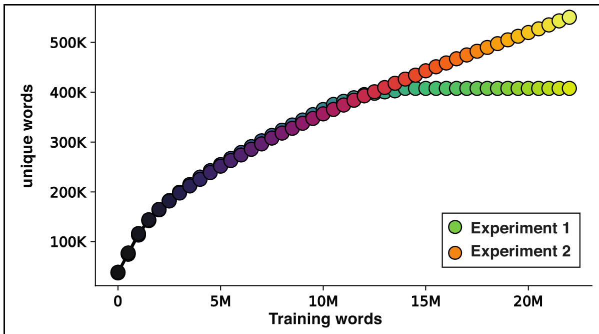

Supplementary Figure 10. Comparison of the number of unique words during the course of training in Experiment 1 vs. Experiment 2. To illustrate the difference between the setup in Experiment 1 vs. 2, we examined the number of unique words relative to the total number of words seen for the first 20M words, in bins of 500K words. For Experiment 1, we focused on the 10M dataset. As can be seen, the green-to-yellow curve reaches a plateau at 10M words, as the model is no longer exposed to new words after that point; instead, the same 10M training dataset is presented again until the best model perplexity is reached. In contrast, the purple-to-yellow curve continues to increase beyond the 10M mark as the model is continually exposed to new words during the course of training (of course, eventually, the increase in new words will become very small because encountering new words becomes less and less likely after exposure to a large enough training corpus).
